# Supplementary material for: Visualization of Germination Proteins in Putative Bacillus cereus Germinosomes
Source: Int J Mol Sci. 2020 Jul 22;21(15):5198. doi: 10.3390/ijms21155198 (PMC7432890; doi:10.3390/ijms21155198)
Supplement: Supplementary file 1 [file ijms-21-05198-s001.pdf]

Visualization of Germination Proteins in Putative *Bacillus cereus* Germinosomes

Table S1. Primers used in this study

Primer names	Primers sequence (5′→3′)	Restriction sites
P_RB-F	CCGGAATTCCACCTTCCCTATATCCGCT[1]	*Eco*R I
P_RB-R	CGGGATCCTCTCACCCCTAACATATATC[1]	*Bam*H I
RB-F	CGGGATCCTGAGGTGAAATGAGCAATGA[1]	*Bam*H I
RB-fuse-GFP-R	CTCGCCCTTGCTCACCAT**GCTGCCGCTGCCGCTGCC**AG GTGTATCGGTTGAAGA[2]	
RB-fuse-GFP-F	TCTTCAACCGATACACCT**GGCAGCGGCAGCGGCAGC** ATGGTGAGCAAGGGCGAG[2]	
GFP-F	GCTCTAGAATGGTGAGCAAGGGCGAG[1]	*Xba* I
GFP-R	CCCAAGCTTTTACTTGTACAGCTCGTCCAT[1]	*Hin*d III
RA-F	CGGGATCCATGTTCGGTTTATCATCT	*Bam*H I
AC-fuse-B-R	CATTGCTCATTTCACCTCACTATTCCCCGATTCCAGATT	
AC-fuse-B-F	AATCTGGAATCGGGGAATAGTGAGGTGAAATGAGCAA TG	
P_D-F	GCTCTAGAACAACCATAAAGAACAGAGC	*Xba* I
D-fuse-mSi-R	TTCTCCTTTACTCACCATGCTGCCGCTGCCGCTGCCCTG TTCTTCCTTCTTCTCG	
D-fuse-mSi-F	CGAGAAGAAGGAAGAACAGGGCAGCGGCAGCGGCAG CATGGTGAGTAAAGGAGAA	
mSi-R	CCGGAATTCTTATTTGTATAGTTCATCCAT	*Eco*R I
315-F	ATGTTGTGTGGAATTGTGAG	
315-R	AAGGCGATTAAGTTGGGT	

[1] The restriction sites are underlined.

[2] The linker sequence are in bold.

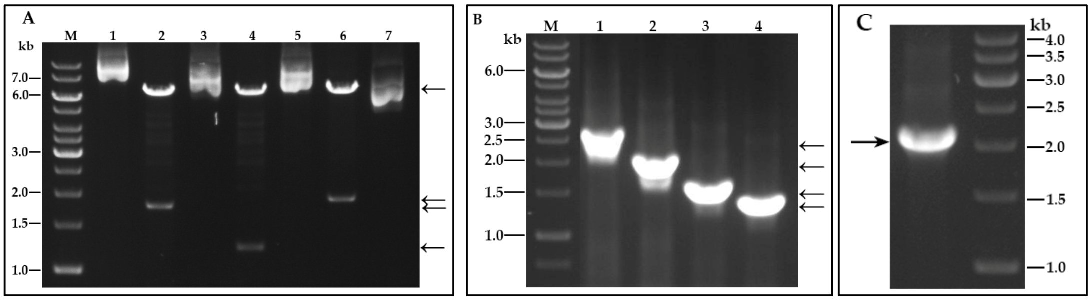

Figure S1. Validation of recombinant plasmids and pHT315-derived *B. cereus* strains. (A) Validation of recombinant plasmids with digestion analysis. Lane M, GeneRuler 1 kb DNA ladder (ThermoFisher Scientific). Lane 1, intact plasmid pHT315-P*gerR-gerRB-SGFP2*; lane 2, the *gerRB-SGFP2* 1.7 kb band and the vector backbone of the 6.5 kb plasmid pHT315-P*gerR-gerRB-SGFP2* cut with *Eco*R I/*Hin*d III; lane 3, intact plasmid pHT315-P*aphA3′-SGFP2*; lane 4, P*aphA3′-SGFP2* 1.2 kb band and the vector backbone of the 6.5 kb plasmid pHT315-P*aphA3′-SGFP2* cut with *Eco*R I/*Hin*d III; lane 5, intact plasmid pHT315-*gerRB-SGFP2*; Lane 6, the *gerRB-SGFP2* of 1.8 kb band and the vector backbone of the 6.5 kb plasmid pHT315-*gerRB-SGFP2* cutting with *Bam*H I/*Hin*d III; lane 7, intact plasmid pHT315. (B) Validation of pHT315-derived *B. cereus* strains. Lane 1, the expected 2.6 kb band was amplified from *B. cereus* carrying pHT315-P*gerR-gerRB-SGFP2* strain; lane 2, the expected 2.0 kb band was amplified from *B. cereus* carrying pHT315-*gerRB-SGFP2* strain; lane 3, the expected 1.4 kb band was amplified from *B. cereus* carrying pHT315-P*gerR-SGFP2* strain; lane 4, the expected 1.3 kb band was amplified from *B. cereus* carrying pHT315-P*aphA3′-SGFP2* strain. (**C**) expected 2.0 kb band in the left lane was amplified from *B. cereus* strain 007. The black arrows indicate the expected bands.

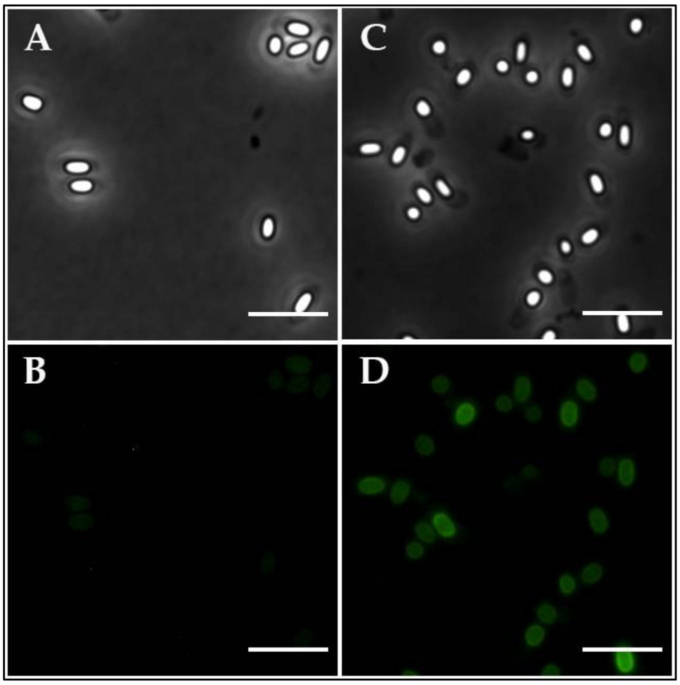

Figure S2. Confirmation of P*gerR* function by examining expression of protein SGFP2 in *B. cereus* dormant spores. Phase-contrast (A, C) and fluorescence microscopy images (B, D) of *B. cereus* dormant spores: (A, B) wild-type spores; (C, D) spores carrying pHT315-P*gerR-SGFP2*. All panels are at the same magnification, and the scale bar is 5 μm.

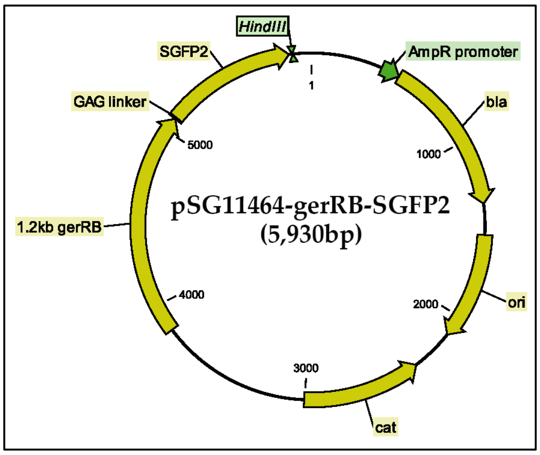

Figure S3. Schematic diagram of integration plasmid pSG1164-*gerRB-SGFP2*. The fusion gene *gerRB-SGFP2* was used to attempt a single crossover integration of the plasmid into the wild-type *gerR* locus in the *B. cereus* chromosome, although this was not successful.
